# Mesenchymal Stem Cell-Like Cells Derived from Mouse Induced Pluripotent Stem Cells Ameliorate Diabetic Polyneuropathy in Mice

**DOI:** 10.1155/2013/259187

**Published:** 2013-11-11

**Authors:** Tatsuhito Himeno, Hideki Kamiya, Keiko Naruse, Zhao Cheng, Sachiko Ito, Masaki Kondo, Tetsuji Okawa, Atsushi Fujiya, Jiro Kato, Hirohiko Suzuki, Tetsutaro Kito, Yoji Hamada, Yutaka Oiso, Kenichi Isobe, Jiro Nakamura

**Affiliations:** ^1^Department of Endocrinology and Diabetes, Nagoya University Graduate School of Medicine, Nagoya 466-8560, Japan; ^2^Department of Immunology, Nagoya University Graduate School of Medicine, Nagoya 466-8560, Japan; ^3^Division of Diabetes, Department of Internal Medicine, Aichi Medical University School of Medicine, Nagakute 480-1195, Japan; ^4^Department of CKD Initiatives, Nagoya University Graduate School of Medicine, Nagoya 466-8560, Japan; ^5^Department of Internal Medicine, School of Dentistry, Aichi-Gakuin University, Nagoya 464-8651, Japan; ^6^Department of Metabolic Medicine, Nagoya University Graduate School of Medicine, Nagoya 466-8560, Japan

## Abstract

*Background*. Although pathological involvements of diabetic polyneuropathy (DPN) have been reported, no dependable treatment of DPN has been achieved. Recent studies have shown that mesenchymal stem cells (MSCs) ameliorate DPN. Here we demonstrate a differentiation of induced pluripotent stem cells (iPSCs) into MSC-like cells and investigate the therapeutic potential of the MSC-like cell transplantation on DPN. *Research Design and Methods*. For induction into MSC-like cells, GFP-expressing iPSCs were cultured with retinoic acid, followed by adherent culture for 4 months. The MSC-like cells, characterized with flow cytometry and RT-PCR analyses, were transplanted into muscles of streptozotocin-diabetic mice. Three weeks after the transplantation, neurophysiological functions were evaluated. *Results*. The MSC-like cells expressed MSC markers and angiogenic/neurotrophic factors. The transplanted cells resided in hindlimb muscles and peripheral nerves, and some transplanted cells expressed S100**β** in the nerves. Impairments of current perception thresholds, nerve conduction velocities, and plantar skin blood flow in the diabetic mice were ameliorated in limbs with the transplanted cells. The capillary number-to-muscle fiber ratios were increased in transplanted hindlimbs of diabetic mice. *Conclusions*. These results suggest that MSC-like cell transplantation might have therapeutic effects on DPN through secreting angiogenic/neurotrophic factors and differentiation to Schwann cell-like cells.

## 1. Introduction

The majority of research on mesenchymal stem cells (MSCs) has focused on their unique ability of their likelihood of differentiating to mesenchymal cells and an immunomodulation [1]. MSCs are obtained from various kinds of tissues: bone marrow, adipose tissue, and umbilical cord blood. Because of this accessibility, MSCs are clinically used for cell-based transplantation therapy for various diseases: stroke, coronary artery disease, graft-versus-host disease, and arteriosclerosis obliterans. The achievements of these cell-based therapies precipitate extended applications in further human diseases. 

In the current study, we deal with diabetic polyneuropathy (DPN). DPN, which is one of the most common peripheral neuropathies as well as one of the most frequent diabetic complications, has not yet been conquered. There is no established therapy for DPN, although many researchers have proven that multiple factors are involved in the pathogenesis: polyol pathway [2], PKC activation [3–6], endoneurial nutritive blood flow [7, 8], advanced glycation end-product formation [9–14], and mitochondrial oxidative stress [15–17]. In the past decade, an increasing number of regenerative therapies, especially cell-based transplantation therapies, in rodent models of DPN have been reported [18–22]. 

Additionally, regenerative research has been accelerated owing to the discovery of induced pluripotent stem cells (iPSC) [23]. iPSCs possess a pluripotency of differentiation into not only terminally differentiated cells but also multipotent somatic progenitor/stem cells [24–26]. Due to the expectancy for clinical applications, MSC-like cells have also been derived from iPSCs [26]. 

Having already indicated the beneficial effects of transplantation of bone-marrow-derived MSCs in diabetic polyneuropathy of type 1 diabetic mice, in this paper, we investigate the effects of cell-based therapy utilizing MSC-like cells derived from mouse iPSCs [18]. Needless to say, this investigation would be the first report of the novel cell therapy, and additionally, this contains two original approaches: one is that we employed iPSC derived from aged mouse with consideration for the feasibility of clinical applications, and another is that endocrine abilities of the obtained MSC-like cells were compared with those of not only mouse MSCs but also mouse Schwann cells.

## 2. Research Design and Methods

### 2.1. Cell Culture

Mouse iPSCs were derived from bone marrow myeloid cells of GFP expressing 21-month-old EGFP-C57BL/6 mice (C14-Y01-FM131Osb), as previously reported [27]. The iPSCs were maintained at 37°C with 5% CO_2_ in DMEM (Life Technologies, Carlsbad, CA) containing 10% Knockout Serum Replacement (Life Technologies), 1% fetal bovine serum (FBS), 1000 U/mL leukemia inhibitory factor (LIF) (EMD Millipore, Billerica, MA), nonessential amino acids, 5.5 mmol/L 2-mercaptoethanol (2ME), 50 U/mL penicillin, and 50 mg/mL streptomycin on feeder layers of mytomycin C-inactivated SNL76/7 cells (the European Collection of Cell Cultures, Salisbury, UK), which were clonally derived from an STO cell line transfected with a G418 resistance cassette and a leukemia inhibitory factor expression construct [28].

### 2.2. Induction of Differentiation into Mesenchymal Stem Cell (MSC)-Like Cells

For the induction into MSC-like cells, iPSCs were maintained in differentiation medium (DM), which is an *α*-minimum essential medium (Life Technologies) supplemented with 10% FBS and 5 × 10^−5^ mol/L 2ME. At day 0 of induction of differentiation, iPSCs were dissociated with the use of trypsin. To promote embryonic body (EB) formation, the cells were cultured at a concentration of 10^3^ cells per 20 *μ*L of DM, utilizing the hanging drop method in petri dishes. After 2 days, the EBs were cultured in DM supplemented with 40 ng/mL of all trans retinoic acid (Sigma-Aldrich Co., St. Louis, MO) for 3 days. The colonies were enzymatically passaged into tissue culture dishes containing the DM and were continuously passaged every 3 or 4 days for 4 months. 

### 2.3. Senescence-Associated Beta-Galactosidase (SA *β*-Gal) Staining

SA *β*-Gal staining was utilized to evaluate cell senescence. MSC-like cells were fixed with 4% PFA and incubated with the staining solution from a Senescence Detection kit (Bio Vision, Mountain View, CA, USA) at 37°C for 24 hours. Stained cells were stored in 70% glycerol and the cells stained light blue were counted under a microscope.

### 2.4. FACS Analysis

The iPSC-derived EBs differentiated by retinoic acid were harvested and incubated in a buffer containing antibodies to selected putative MSC surface antigens. The antibodies used for FACS analysis were phycoerythrin (PE) conjugated to anti-CD11b, anti-CD31, anti-CD34, anti-CD44, anti-CD45, anti-TER119, anti-PDGFR*α*, anti-Sca-1, and unconjugated antibodies against CD105 (BD Biosciences, San Diego, CA). A total of 2 × 10^5^ cells from different treatments were resuspended in 200 *μ*L of Dulbecco's PBS containing 2% FBS and incubated for 30 minutes at 4°C with the antibodies. The cells incubated with the anti-CD105 antibody were followed by PE-conjugated secondary antibodies (Santa Cruz Biotechnology Inc., Santa Cruz, CA). The proper isotype-identical immunoglobulins served as controls. After staining, the cells were fixed in 2% paraformaldehyde, and quantitative FACS analysis was performed on a FACSCalibur flow cytometer (BD Biosciences, San Diego, CA). 

### 2.5. Real-Time Reverse Transcription PCR (RT-PCR) Analysis

Total RNA was isolated from cultured MSC-like cells. RNA was reverse transcribed into cDNA by ReverTraAceqPCR RT kit (Toyobo, Osaka, Japan) according to the manufacturer's instructions. Primers were designed by Primer3 software (http://frodo.wi.mit.edu/) and tested for specificity with NCBI-BLAST (http://www.ncbi.nlm.nih.gov/tools/primer-blast/). Primer sequences are shown in Table 1. Real-time quantitative PCR was performed and monitored using the Mx3000P QPCR System (Stratagene Agilent Technologies, Santa Clara, CA) with SYBR Green I as a double-stranded DNA-specific dye according to the manufacturer's instructions (Applied Biosystem, Foster City, CA). The PCR products were analyzed with agarose gel containing ethidium bromide to confirm these predicted lengths. Relative quantity was calculated using the ΔΔCt method. 

### 2.6. Cell Differentiation into Osteocyte, Chondrocyte, and Adipocyte

The MSC-like cells were differentiated as previously reported [29]. For the induction to osteoblast, MSC-like cells were seeded at 1 × 10^5^ cells/cm^2^ in DMEM supplemented with 10% fetal calf serum (FCS), 100 nM dexamethasone (Sigma-Aldrich), 2 mM *β*-glycerophosphate (Sigma-Aldrich), and 150 *μ*M ascorbate-2-phosphate (Sigma-Aldrich). Cells were incubated until the 21st day, with a medium change every 4th day. After the induction, alkaline phosphatase staining was performed on the cells utilizing a Leukocyte Alkaline Phosphatase Kit (Sigma-Aldrich).

For the induction into chondrocyte, 3 × 10^5^ MSC-like cells were centrifuged in a 48 well plate to form a pellet and incubated for 21 days in 500 *μ*L of DMEM with 170 *μ*M ascorbic acid-2-phosphate, 100 nM dexamethasone, 350 *μ*M proline, 1 × insulin-transferrin-selenium (Gibco), 1 mM sodium pyruvate (Sigma-Aldrich), and 10 ng/mL transforming growth factor-*β*1 (Sigma-Aldrich). The medium was changed every 4 days. For Alcian blue staining, the pellets were fixed with 4% PFA for 8 hours and then embedded in O.C.T. compound (Sakura Finetechnical, Tokyo, Japan). The embedded blocks were sectioned and incubated with 10% Alcian blue (Sigma-Aldrich) in 0.1 N HCl for 30 minutes.

Adipogenic differentiation was induced by culturing in DMEM supplemented with 20% FCS, 1 *μ*M dexamethasone, 350 nM hydrocortisone, 500 *μ*M isobutyl-methylxanthine (Sigma-Aldrich), 100 ng/mL insulin (Sigma Aldrich), and 60 *μ*M indomethacin (Sigma-Aldrich). Cells were seeded at 2 × 10^4^ cells/cm^2^ and cultured for 12 days at 37°C. The medium was changed every 4 days. For Oil Red-O staining, the cells were fixed with 4% PFA and stained with Oil Red-O (Sigma-Aldrich) dissolved in 60% isopropyl alcohol for 15 minutes. 

### 2.7. Western Blotting

The MSC-like cells were lysed in detergent lysis buffer (Cell Lysis Buffer, Cell Signaling Technology, Boston, MA) supplemented with 1 mM phenylmethanesulfonylfluoride (Sigma-Aldrich), followed by centrifugation. After the concentration of proteins was measured with a BCA assay (Sigma Chemical), the proteins were transferred to polyvinylidene fluoride membranes (Millipore, Billerica, MA) after SDS-PAGE. The membranes were blocked and incubated with rabbit anti-p19 and anti-p16 antibodies (Santa Cruz Biotechnology Inc., Santa Cruz, CA). Antigen detection was performed using ECL Plus Western Blotting Detection Reagents (Amersham Pharmacia Biotech, Piscataway, NJ) with horseradish peroxidase-conjugated anti-rabbit IgG antibody (Cell Signaling Technology). Images were scanned and their densities were determined by ImageJ (National Institutes of Health, Bethesda, MD). The expressions of the proteins were corrected by beta-actin density, and the expression in tissues of normal mice was arbitrarily set at 1.0.

### 2.8. Animals and Induction of Diabetes

Five-week-old male C57BL/6 mice (Chubu Kagaku Shizai, Nagoya, Japan) were allowed to adapt to the experimental animal facility for 7 days. The animals were housed in an aseptic animal room under controlled light/dark and temperature conditions with food and water available ad libitum. Diabetes was induced by intraperitoneal injection (i.p.) of streptozotocin (STZ) (150 mg/kg; Sigma-Aldrich). Control mice received an equal volume of citric acid buffer. One week after STZ administration, the mice with plasma glucose concentrations of >16 mmol/L were classified as diabetic group. Twelve weeks after the induction of diabetes, 1 × 10^5^ cells/limb of the MSC-like cells in 0.2 mL saline were injected into the right thigh and soleus muscles of normal and diabetic mice. The corresponding left hindlimb muscles were treated with saline alone. Three weeks later, the mice were harvested after evaluation of hemodynamic and neurophysiological parameters: blood flow of plantar skin, current perception threshold (CPT), nerve conduction velocity (NCV), and the thermal plantar test (TPT). Before the transplantation of the MSC-like cells, fasting blood glucose levels and hemoglobin A1c were examined by a FreeStyle Freedom Glucose Meter (Nipro, Osaka, Japan) and a RAPIDIA Auto HbA1c-L assay kit, using latex agglutination (Fujirebio Inc., Tokyo, Japan), respectively. The Nagoya University Institutional Animal Care and Use Committee approved the protocols of this experiment. 

### 2.9. Measurement of CPTs Using a Neurometer

To determine a nociceptive threshold, CPTs were measured in 12- and 15-week diabetic and age-matched nondiabetic mice using a CPT/LAB neurometer (Neurotron, Denver, CO) according to the method by Shibata et al. [18] with minor modifications. The electrodes (SRE-0405-8; Neurotron) for stimulation were attached to plantar surfaces of the mice. Each mouse was kept in a Ballman cage (Natsume Seisakusho, Tokyo, Japan) suitable for light restraint to keep the mice awake. Three transcutaneous-sine-wave stimuli with different frequencies (2000, 250, and 5 Hz) were applied to the plantar surfaces of the mice to determine the CPT of sensory perceptions (pressure, pain, and pain and temperature, respectively). The intensity of each stimulation was gradually and automatically increased (increments of 0.01 mA for 5 and 250 Hz, increments of 0.02 mA for 2000 Hz). The minimum intensity at which each mouse withdrew its paw was defined as the CPT. Six consecutive measurements were conducted at each frequency.

### 2.10. NCV

After intraperitoneal injection of sodium pentobarbital (5 mg/100 g), mice were placed on a heated pad in a room maintained at 25°C to ensure a constant rectal temperature of 37°C. Motor NCV (MNCV) was calculated between the sciatic notch and the ankle using a Neuropak NEM-3102 instrument (Nihon-Koden, Osaka, Japan), as previously described [18, 30, 31]. The sensory NCV (SNCV) was measured between the hindlimb knee and ankle using retrograde stimulation.

### 2.11. TPT

Three weeks after the transplantations, hind paw withdrawal response against thermal stimuli of radiant heat was measured using a plantar test 7370 device (Ugo Basile, Comerio, Italy). Radiant heat was beamed onto the plantar surface of the hind paw. The paw withdrawal latencies were measured six times per session, separated by a minimum interval of 5 minutes. Paw withdrawals due to locomotion or weight shifting were not counted. Data are expressed as paw withdrawal latency in seconds. 

### 2.12. Blood Flow of Plantar Skin

Before the evaluation of the MNCV and SNCV, blood flow of the plantar skin was measured by a laser Doppler probe (laser-Doppler flowmetry FLO-N1; Omegawave Inc., Tokyo, Japan) placed 1 mm above the skin. During this measurement, the mouse was placed on a heated pad in a room maintained at 25°C to ensure a constant rectal temperature of 37°C.

### 2.13. Histochemistry

Three weeks after the transplantation, the mice were anesthetized with sodium pentobarbital (5 mg/100 g) and perfused with 20 ml of 4% PFA fixative. After perfusion, soleus muscles with a sural nerve were excised and fixed in 4% PFA at 4°C overnight. Specimens were immersed in PBS containing 20% sucrose, embedded within an O.C.T. compound (Sakura Finetechnical) and cut into 6 *μ*m sections, which were used for staining as previously reported [18, 19] with minor modifications. The vascular capillaries were stained by Alexa Fluor 594 conjugate isolectin GS-IB4 (Life Technologies) and were counted under a fluorescence microscope (BX51, Olympus Optical) and images were obtained by a CCD camera (DP70, Olympus Optical). Five fields from each section were randomly selected for the capillary counts. GFP-expressing cells representing the transplanted MSC-like cells were observed under a microscope. Nuclei were stained with DAPI (Merck).

### 2.14. Statistical Analysis

All of the group values were expressed as means ± SD. Statistical analyses were made by one-way ANOVA, with the Bonferroni correction for multiple comparisons. All analyses were performed by personnel unaware of the animal identities.

## 3. Results

### 3.1. The Aging Markers in MSC-Like Cells Obtained from Aged-Mouse iPSC Decreased with Time

**Figure 1:**
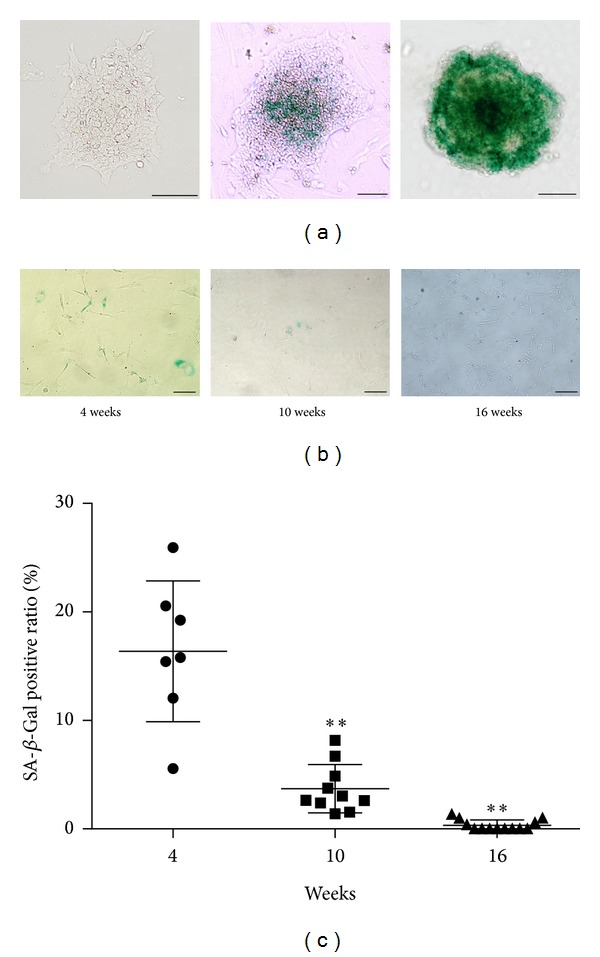
Senescence-associated beta-galactosidase (SA *β*-Gal) staining. (a) The induced pluripotent stem cell colonies obtained from aged mice colonies were heterogeneously expressed with SA *β*-Gal (green). Scale bar = 50 *μ*m. (b) After long term culture, differentiated MSC-like cells expressing SA *β*-Gal (green) decreased. Scale bar = 100 *μ*m. (c) The ratio of SA *β*-Gal positive mesenchymal stem cell (MSC)-like cells decreased significantly in 10 weeks or 16 weeks cultured MSC-like cells compared with 4 weeks cultured cells. 4 wks: the MSC-like cells cultured for 4 weeks, 10 wks: the MSC-like cells cultured for 10 weeks, and 16 wks: the MSC-like cells cultured for 16 weeks. **P < 0.005 compared with 4 wks.

The iPSCs derived from a 21-month-old mouse contained the aging marker SA *β*-Gal. However, the levels of SA *β*-Gal varied among iPS cell colonies (Figure 1(a)). After a 16-week culture, MSC-like cells kept their fibroblast-like morphology, adherent to a dish bottom and flatly spread, and the ratio of SA *β*-Gal positive cells decreased over time (Figures 1(b) and 1(c)).

**Figure 2:**
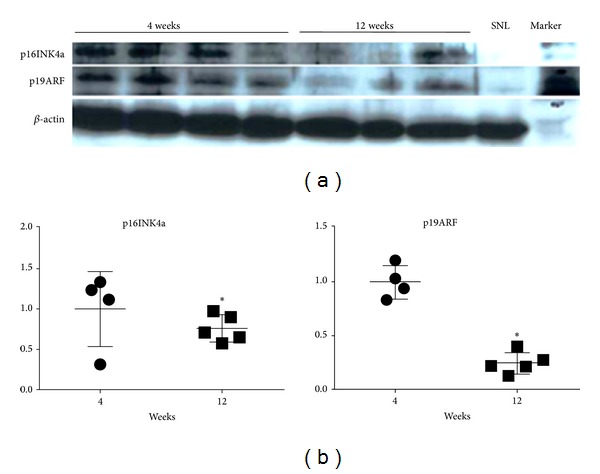
The protein expression levels of p16INK4a and p19ARF. (a) Upon Western blotting, the senescence marker proteins, p16INK4a and p19ARF, were expressed in the mesenchymal stem cell (MSC)-like cells after 4 weeks culture. Both levels decreased in the cells after 12 weeks culture. (b) In a densitometric analysis with correction of *β*-actin intensity, both protein levels in the cells cultured for 12 weeks decreased significantly compared with the levels in the 4-week cultured cells. 4 wks: the MSC-like cells cultured for 4 weeks, 10 wks: the MSC-like cells cultured for 10 weeks, *P < 0.05 compared with 4 wks.

Although p16INK4a and p19ARF, other markers of cell aging, were highly expressed in the early stages of the culture compared with immortalized SNL feeder cells (Figure 2(a)), the expression levels of p19ARF significantly decreased and those of p16INK4a presented a reduction tendency after 3 months (Figures 2(a) and 2(b)). 

### 3.2. The MSC-Like Cells Expressed MSC Markers and Differentiated into Mesenchymal Derived Cells

**Figure 3:**
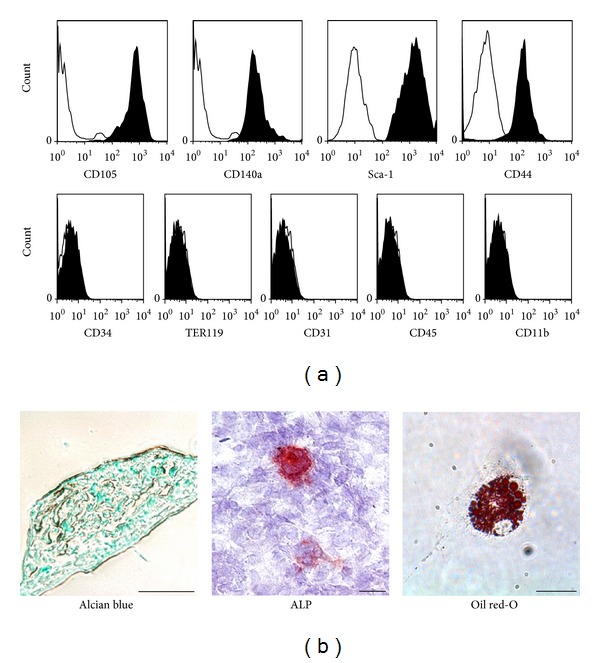
The characterization of the mesenchymal stem cell (MSC)-like cells. (a) Flow cytometry analyses of mesenchymal stem cell (MSC) and hematopoietic cell lineage markers in the MSC-like cells. The MSC-like cells expressed MSC markers, CD105, CD140a, Sca-1, and CD44, in spite of no expression of hematopoietic markers, CD34, TER119, CD31, CD45, and CD11b. Open curves: control, filled curves: each of target antibodies. (b) After differentiation of the MSC-like cells *in vitro*, each differentiated cells into chondrocyte, osteoblast, and adipocyte exhibited staining abilities with Alcian blue, alkaline phosphatase, and Oil Red-O, respectively. ALP: alkaline phosphatase staining, scale bar = 50 *μ*m.

The MSC-like cells expressed cell surface markers of MSC, that is, CD105, CD140a, Sca-1, and CD44, and expressed no haematopoietic lineage markers, that is, CD34, TER119, CD31, CD45, and CD11b (Figure 3(a)). After their induction into three mesenchymal derived cells, that is, chondrocyte, osteoblast, and adipocyte, each of the differentiated cells was stained with specific dyes. The cells induced into chondrocytes exhibited stainability with Alcian blue, the cells into osteoblast were confirmed their alkaline phosphatase activity, and the cells into adipocytes were proven that they contained lipid droplets within their cytoplasm utilizing Oil Red O staining (Figure 3(b)). 

### 3.3. MSC-Like Cells Expressed Angiogenic and Neurotrophic Factors

**Figure 4:**
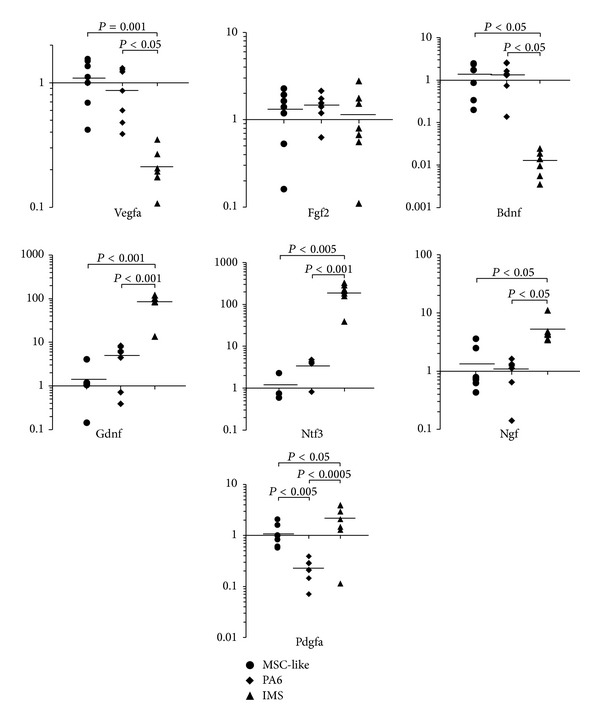
The expression ability of growth factors in the mesenchymal stem cell (MSC)-like cells. Transcript levels of growth factors were examined in the MSC-like cells, mouse MSC cell-line PA6, and mouse immortalized Schwann cell-line IMS. Each data was presented as a fold change of each expression level in the MSC-like cells. Filled circle: the MSC-like cells, filled diamond shape: PA6, filled triangle: IMS. *n* = 5 in each group.

To verify the endocrine effect of the MSC-like cells, the following mRNA expression levels of angiogenic and neurotrophic factors were evaluated in the cells: VEGF-A, PDGF-A, FGF2, NGF, brain-derived neurotrophic factor (BDNF), glial cell line-derived neurotrophic factor (GDNF), Neurotrophin-3 (NT-3), and ciliary neurotrophic factor (CNTF) (Figure 4). For comparison, immportalized mouse MSC cell-line PA6 cells were evaluated. Furthermore, because Schwann cells have been established as an intrinsic neuroprotective cell, the endocrine ability of IMS cells, immortalized mouse Schwann cells, was also compared to that of MSC-like cells. The MSC-like cells indicated a similar pattern of endocrine ability as PA6 MSC except for PDGF-A, in which the MSC-like cells expressed significantly high transcript levels compared to PA6. Compared to the IMS cell, the MSC-like cells expressed a comparable level of FGF2, higher levels of VEGF-A and BDNF, and lower levels of GDNF, NT3, NGF, and PDGF-A (Figure 4).

### 3.4. Body Weight and Blood Glucose Levels

Twelve weeks after the STZ injection, the diabetic mice showed severe hyperglycemia and significantly reduced body weight gain, and after the transplantation, there was no significant change in either group (Table 2).

### 3.5. The Transplanted Cells Were Found within Skeletal Muscles and Peripheral Nerves

**Figure 5:**
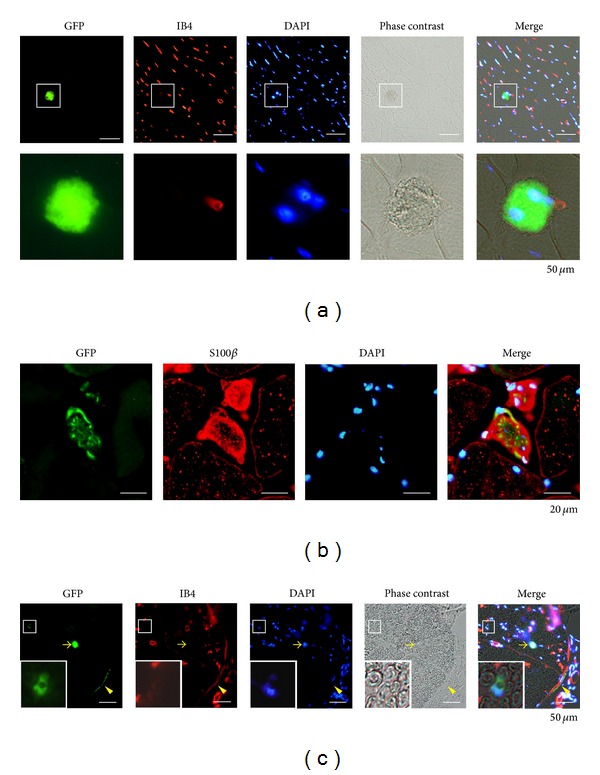
Distribution of the transplanted mesenchymal stem cell (MSC)-like cells. (a) The transplanted MSC-like cells expressing GFP (inset) engrafted in a soleus muscle (upper panels). Lower panels are magnified images of insets. Scale bar = 50 *μ*m. IB4: isolectin GS-IB4. (b) In a soleus muscle, the MSC-like cells engrafted in one of the peripheral nerve branch and expressed S100*β*, a Schwann cell marker. Scale bar = 20 *μ*m. (c) The GFP expressing MSC-like cells (yellow arrow and inset) engrafted in a sural nerve. Some engrafted cells surrounded a nerve fiber (inset). Some engrafted cells resided close to a perineurium (yellow arrow head). Scale bar = 50 *μ*m. IB4: isolectin GS-IB4.

Two months after the transplantation, some treated mice were harvested to verify the engraftment of the GFP-expressing (GFP^+^) transplanted cells within the tissues of the recipients. No teratoma was detected in the rough sectioned tissue slices of the soleus muscles, brains, hearts, lungs, or livers, and GFP^+^ cells were nonexistent except in the muscles and nerves of the transplanted hindlimbs (data not shown). Some GFP^+^ cells, which resided among muscle fibers, appeared not to form any functional tissue structure (Figure 5(a)), and the other GFP^+^ cells, residing within or around peripheral nerves, expressed S100*β*, one of the Schwann cell markers (Figures 5(b) and 5(c)). A relationship between vessels and GFP^+^ engrafted cells was not obviously observed in sural nerves (Figure 5(c)).

### 3.6. The Blood Flow of Plantar Skin and the Capillary Number to Muscle Fiber Ratios Increased in Treated Limbs

**Figure 6:**
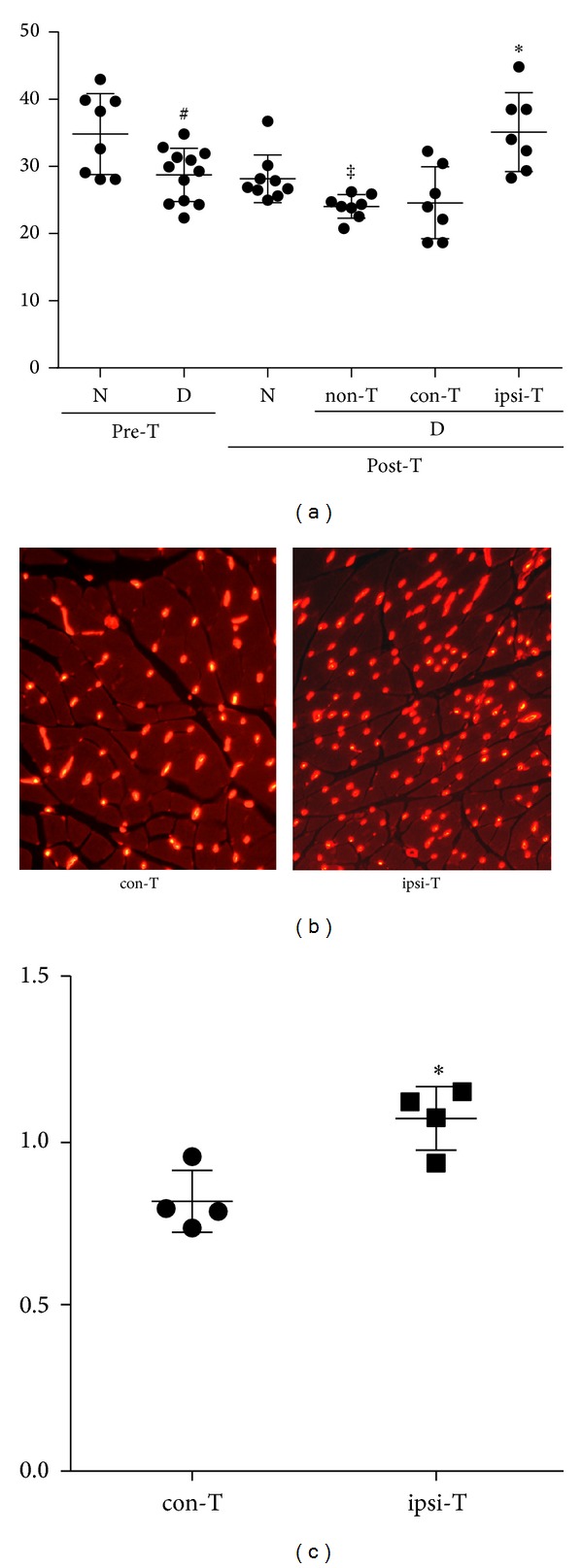
Plantar skin blood flow and capillary number in a soleus muscle. (a) Plantar skin blood flow in diabetic mice significantly decreased compared with that in nondiabetic mice. The flow was ameliorated in transplanted mice. ^#^ *P* < 0.05 compared with pretransplanted nondiabetic mice, ^‡^ *P* < 0.05 compared with posttransplanted mice, *P < 0.05 compared with non-T. D: diabetic mice, N: nondiabetic mice, con-T: contralateral limbs of transplanted mice, ipsi-T: ipsilateral limbs of transplanted mice. *n* = 7–12 in each group. (b, c) In a soleus muscle, capillaries were visualized with isolectin GS-IB4 (red). Quantification of the capillary-to-muscle number ratio revealed the increase of the ratio in transplanted limbs. *P < 0.05 compared with con-T. con-T: contralateral limbs of transplanted mice, ipsi-T: ipsilateral limbs of transplanted mice. *n* = 4 in each group.

By 12 weeks after the onset of diabetes, the blood flow of plantar skin in diabetic mice decreased significantly compared with that in nondiabetic mice, but the decrease was ameliorated in the limbs treated with MSC-like cells (Figure 6(a)).

The vasculatures were visualized by Alexa594-conjugated isolectin IB4, a marker for endothelial cells (Figure 6(b)). Transplantation of MSC-like cells significantly augmented the capillary number to muscle fiber ratios in the transplanted limbs (ipsi-T) compared with the ratio in the saline-injected side limbs (con-T) in diabetic mice (Figures 6(b) and 6(c)).

### 3.7. Reduced Sensory Perception in Diabetic Mice Was Ameliorated by the MSC-Like Cell Transplantation

**Figure 7:**
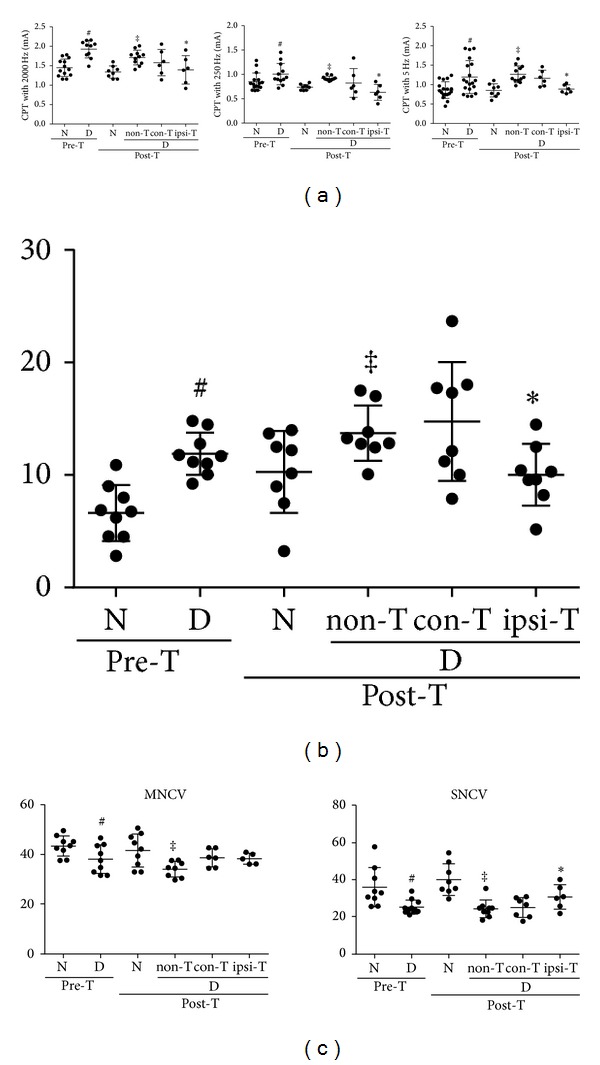
Neurophysiological evaluations. (a) All of current perception thresholds (CPTs) were impaired in diabetic mice and the impairments were ameliorated in the transplanted limbs. (b) The thermal plantar test clarified the impairments of thermal perception in diabetic mice and the impairments were ameliorated in the transplanted limbs. (c) Motor and sensory nerve conduction velocity (MNCV and SNCV, respectively) decreased in diabetic mice compared with those in nondiabetic mice. The SNCV increased in the transplanted limbs of diabetic mice. ^#^ *P* < 0.05 compared with pretransplanted nondiabetic mice, ^‡^ *P* < 0.05 compared with posttransplanted mice, *P < 0.05 compared with non-T. D: diabetic mice, N: nondiabetic mice, con-T: contralateral limbs of transplanted mice, and ipsi-T: ipsilateral limbs of transplanted mice. *n* = 6–19 in each group.

After 12 weeks of diabetes, current perception thresholds (CPTs) at 5, 250, and 2000 Hz had significantly increased compared with those in normal mice, representing hypoalgesia in diabetic mice. Three weeks after the transplantation, these deficits in sensation had significantly improved in diabetic mice compared with saline-treated diabetic controls (Figure 7(a)). To strengthen the existence of the perception dysfunction, TPT was performed. The actual perception of thermal stimuli was also impaired in diabetic mice after the 12-week diabetic duration, and, consistent with the result of CPT, the impairment was also ameliorated in the transplanted limbs (Figure 7(b)).

### 3.8. MSC-Like Cells Improved Delayed SNCV in Diabetic Mice

Motor nerve conduction velocity and sensory nerve conduction velocity of diabetic mice were significantly delayed compared with those of normal mice (Figure 7(c)). The delay of SNCV was significantly restored in the transplanted limbs three weeks after the treatment.

## 4. Discussion

The present study demonstrated that MSC-like cells could be obtained from aged mouse iPSCs and the transplantation of the MSC-like cells ameliorated physiological impairments and reduced blood flow of plantar skin in DPN. The histological appearance revealed that the capillary number to muscle fiber ratios increased in the skeletal muscles of the transplanted lower limbs. Furthermore, the transplanted cells were grafted around the injected sites, and some of them differentiated to S100*β* expressing cells in peripheral nerves.

MSCs, which might contain heterogeneous subpopulations of cells, are generally characterized as having the ability of differentiation into chondrocytes, osteoblasts, and adipocytes, and have no cell surface markers of hematopoietic cell lineages [32]. In addition, they adhere to the bottom of tissue culture dishes and expand into the shape of fibroblast-like spindle cells. In this study, we acquired adherent spindle cells and demonstrated their *in vitro* multilineage differentiation abilities and their lack of hematopoietic lineage markers. As a cell surface marker specific to mouse MSCs remains undefined, we examined a combination of markers, that is, CD105, CD140a, Sca-1, and CD44, according to a previous paper [33], and confirmed the existence of these markers on the MSC-like cells.

In spite of widespread research on MSCs, the physiological roles of MSCs in adult animals and humans have not been elucidated sufficiently. Therefore, most transplantation therapies of MSCs are expected to exert their immunosuppressive properties and cytoprotection or tissue regeneration through their paracrine effects [34–36]. As we employed the MSC-like cells in anticipation of their paracrine effect, the transcript levels of trophic growth factors in these cells were assayed and compared with those in murine cell-lines of a MSC and a Schwann cell, which supports peripheral neurons physically and chemically. The transcript levels of MSC-like cells were comparable to those of the MSC cell-line, while the expression pattern in the MSC-like cells was distinguished from the pattern in the Schwann cell-line. Experiments comparing cell therapy using Schwann cell precursors with therapy using MSC-like cells could be inspected in the future.

In general, stem cells acquired from aged animals also display phenomena of cell senescence, and many researchers have tried to achieve cell rejuvenation [37, 38]. Recent papers have indicated that reprograming somatic cells into pluripotent cells might facilitate the rejuvenation [39, 40]. Consistent with these previous reports, we presented the reduction of cell senescence related proteins, that is, SA-*β*-Gal, p16INK4a, and p19ARF, in the long-cultured reprogrammed cells. However, these proteins were not suppressed immediately after the reprogramming. We speculate from the heterogeneous staining properties of the iPS cell colonies with SA-*β*-Gal that incompletely reprogrammed iPSCs would not proliferate and be negatively selected through the lengthy culturing period, and only completely reprogrammed iPSCs would expand. Further experiments including telomerase assay and DNA methylation analysis to confirm the accuracy of cell senescence should be developed in the future. 

Although our original purpose was to investigate the paracrine/endocrine effect of MSC-like cells in DPN, the MSC-like cells engrafted in peripheral nerves, surpassed our expectation, by expressing S100*β*, a Schwann cell marker. This fact could indicate that the grafted cells might directly construct peripheral nervous tissues. Although it has been reported that MSCs differentiated to Schwann cell-like cells *in vitro* [41], *in vivo* differentiation of MSCs into Schwann cells has not yet been documented. On the other hand, cell fusion between proinsulin-producing bone marrow-derived cells and dorsal root ganglion neurons in DPN has been described [42]. Therefore, further experiments to exclude the possibility of cell fusion and to elucidate the Schwann cell function of the grafted cells should be considered.

In conclusion, we have demonstrated the beneficial effects of transplantation of MSC-like cells derived from iPSCs on DPN. Although additional studies to reveal the safety of the transplantation on DPN would be required, this cell transplantation appears to be a novel therapeutic strategy for DPN. 

## Figures and Tables

**Table 1:** Primer sequences.

Accession number	Gene	Forward primer (5′ → 3′)	Reverse primer (3′ → 5′)
NM_001025250.3	Vegfa	CAGGCTGCTGTAACGATGAA	TTTCTTGCGCTTTCGTTTTT
NM_008006.2	Fgf2	GTGGATGGCGTCCGCGAGAA	ACCGGTTGGCACACACTCCC
NM_008808.3	Pdgfa	GAGATACCCCGGGAGTTGAT	TCTTGCAAACTGCAGGAATG
NM_001048139.1	Bdnf	GCCACCGGGGTGGTGTAAGC	CATGGGTCCGCACACCTGGG
NM_001112698.1	Ngf	GTGAAGATGCTGTGCCTCAA	GCGGCCAGTATAGAAAGCTG
NM_010275.2	Gdnf	CGGACGGGACTCTAAGATGA	CGTCATCAAACTGGTCAGGA
NM_001164034.1	Ntf3	CGAACTCGAGTCCACCTTTC	AGTCTTCCGGCAAACTCCTT

**Table 2:** Body weights and blood glucose levels.

	Non-diabetic mice		Diabetic mice	
	Pretransplantation	Posttransplantation	Pretransplantation	Posttransplantation
Number	10	10	8	8
Blood glucose (mmol/L)	9.1 ± 1.5	8.2 ± 1.6	23.1 ± 2.8*	22.3 ± 2.3^#^
Body weight (g)	30.6 ± 2.7	31.3 ± 3.0	26.1 ± 1.0*	28.3 ± 0.7^#^

Results are means ± SD. *P < 0.05 versus pretreatment non-diabetic mice. ^#^ *P* < 0.05 versus posttreatment non-diabetic mice.
